# Process modeling, techno-economic assessment, and life cycle assessment of the electrochemical reduction of CO_2_: a review

**DOI:** 10.1016/j.isci.2021.102813

**Published:** 2021-07-01

**Authors:** Ana Somoza-Tornos, Omar J. Guerra, Allison M. Crow, Wilson A. Smith, Bri-Mathias Hodge

**Affiliations:** 1 Renewable and Sustainable Energy Institute, University of Colorado, Boulder, CO 80309, USA; 2 National Renewable Energy Laboratory, Golden, CO, USA

**Keywords:** Electrochemistry, Energy resources, Energy policy, Energy engineering, Energy sustainability, Energy Systems

## Abstract

The electrochemical reduction of CO_2_ has emerged as a promising alternative to traditional fossil-based technologies for the synthesis of chemicals. Its industrial implementation could lead to a reduction in the carbon footprint of chemicals and the mitigation of climate change impacts caused by hard-to-decarbonize industrial applications, among other benefits. However, the current low technology readiness levels of such emerging technologies make it hard to predict their performance at industrial scales. During the past few years, researchers have developed diverse techniques to model and assess the electrochemical reduction of CO_2_ toward its industrial implementation. The aim of this literature review is to provide a comprehensive overview of techno-economic and life cycle assessment methods and pave the way for future assessment approaches. First, we identify which modeling approaches have been conducted to extend analysis to the production scale. Next, we explore the metrics used to evaluate such systems, regarding technical, environmental, and economic aspects. Finally, we assess the challenges and research opportunities for the industrial implementation of CO_2_ reduction via electrolysis.

## Graphical Abstract

**Figure:**
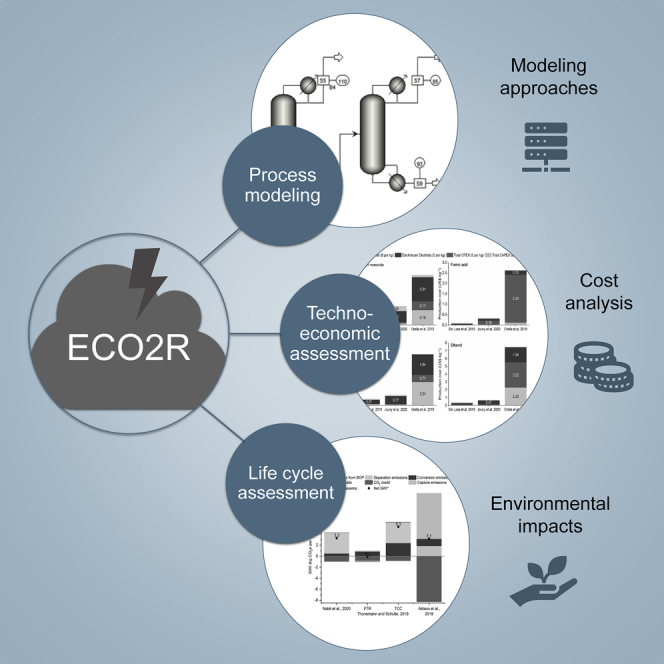

## Introduction

In recent years, carbon capture and utilization (CCU) technologies have emerged as key components of carbon mitigation pathways to decarbonize hard-to-abate sectors (e.g., shipping, aviation, and industrial applications). Indeed, the past decade has seen rapid progress in research and development in CCU technologies in the search for recarbonization pathways for industrial and chemical processes (Birdja et al., 2019; De Luna et al., 2019; Schiffer and Manthiram, 2017). The main motivation behind CO_2_-based chemistry is not to remediate CO_2_ emissions but to decarbonize the synthesis of chemicals by providing cleaner alternatives to fossil-based precursors (Artz et al., 2018; Babacan et al., 2020). CCU pathways may include processes at different fundamental chemistry pathways: biochemical, bioelectrochemical, electrochemical, photocatalytic, photosynthetic, and thermo-catalytic processes. Each alternative shows strengths and weaknesses in different areas. A coordinated effort toward their cost-effective integration into the process chain and energy systems will be needed to drive the shift toward a low carbon economy, which will require the integration of carbon neutral energy sources into the oil and gas and other chemicals sectors, which make up 6.2% and 0.3% of current direct carbon emissions in the United States, respectively (United States Environmental Protection Agency, 2019).

Energy systems around the world are evolving toward more integrated, cleaner, and sustainable processes. However, achieving a carbon-free economy is a daunting task, as it requires significantly reducing emissions from difficult-to-decarbonize sectors, including industrial and chemical processes (Davis et al., 2018; Hepburn et al., 2019). On the other hand, recent and rapid progress in renewable power generation technologies, e.g., wind and solar photovoltaic power, could facilitate the transition from fossil-based to renewable-based energy systems (Chu et al., 2017; Haegel et al., 2019; Veers et al., 2019). However, these renewable energy technologies have variable output at both daily and seasonal scales, leading to times of both shortages and surpluses. This presents interesting opportunities for flexible Power-to-X technologies which can both increase flexible electricity demand and potentially provide a new fuel source that can shift electricity production temporally and/or spatially, as well as providing decarbonization pathways for other sectors. Due to the wide variety of both potential products and applications, the consolidation of a roadmap for the industrial implementation of renewable-based CCU requires a cross-sectoral systems engineering approach (Grim et al., 2020).

In this direction, the electroreduction of CO_2_ into chemicals (ECO2R) is a technology with the potential to produce valuable products and use excess renewable energy but presents major economic and performance challenges in terms of efficiency, flexibility, and durability (Martín et al., 2015). To become a disruptive technology and displace or compliment petrochemical processes, ECO2R is expected to yield multi-carbon products (i.e. C_2+_ products) as one means of increasing capital utilization and hence revenue. However, due to the current state of the technology, single-carbon products present the most economically compelling targets (Bushuyev et al., 2018). At earlier stages of implementation, quantitative methods for the assessment of ECO2R processes become crucial to guide research based on technical, economic, and environmental targets. Process modeling, techno-economic assessment, and life cycle assessment (LCA) of emerging technologies are both a key instrument and a major challenge for ECO2R assessment and decision-making. Some of these aspects have been the subject of research efforts from the general perspective of CCU (Artz et al., 2018; Centi et al., 2020; Thonemann, 2020).

In this work, we aim to provide a comprehensive overview of the modeling and assessment of the electroreduction of CO_2_ into valuable chemicals. The main issues addressed in this review are (a) the modeling approaches that are implemented to bridge the information gap between the laboratory and the production scale, (b) the metrics used to evaluate ECO2R technologies, regarding performance, environmental, and economic aspects, and (c) the challenges and research opportunities for the industrial implementation of ECO2R.

## Methods

**Figure 1:**
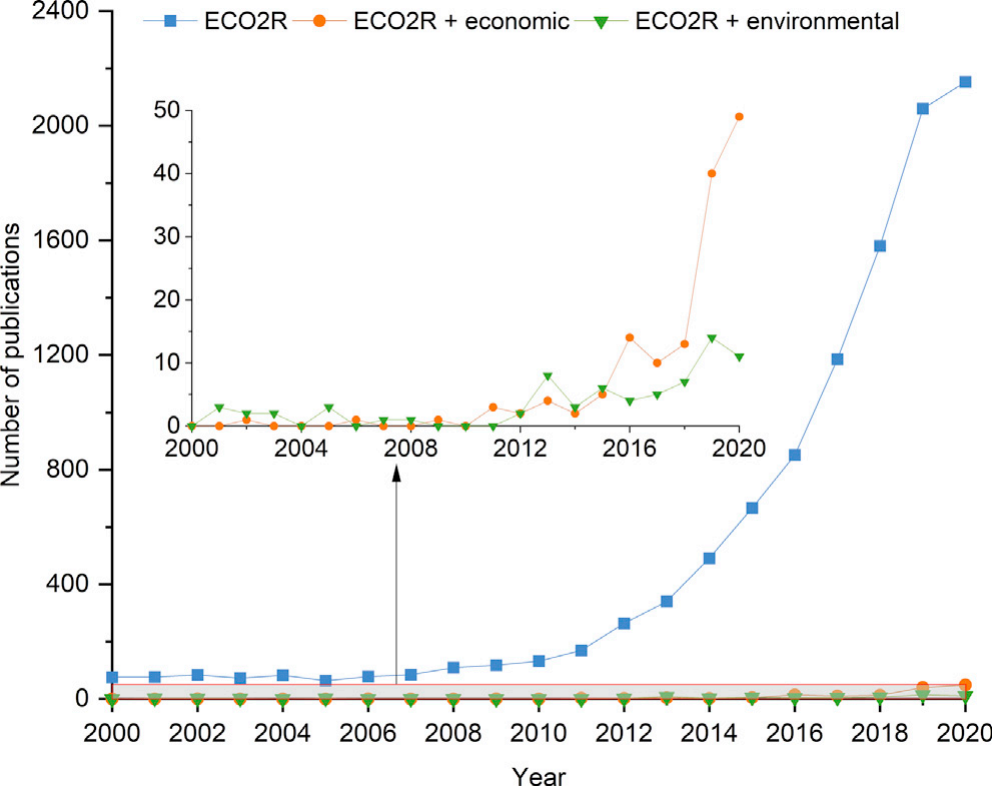
Literature overview Number of contributions within years 2000–2020 resulting from the query terms “electro∗ and (∗reduction near CO2)” (ECO2R), “electro∗ and (∗reduction near CO2) and economic” (ECO2R + economic) and “electro∗ and (∗reduction near CO2) and (environmental NEAR (assessment OR analysis OR impact))” (ECO2R + environmental)

With the purpose of assessing the state of the art of ECO2R in a systematic manner, we used the Web of Science search engine to search for the query: electro∗ and (∗reduction near CO_2_). Note that the asterisks and “*near”* operator are used to include alternative terminology used to refer to this technology (e.g., CO_2_ reduction, electroreduction of CO_2_, electrosynthesis, etc.). This gives a total of 10,738 articles published in peer-reviewed journals as of January 2021. Additionally, we refined the search to pinpoint quantitative methods for the economic assessment of ECO2R processes. When the term “economic” is added to the search query to identify the contributions with economic considerations, it results in a subset of 145 peer-reviewed journal papers. Likewise, 72 documents were found after filtering the results that satisfied the query “(environmental NEAR (assessment OR analysis OR impact))”. Figure 1 shows the trends in the number of contributions resulting from these three queries within the last two decades.

As indicated by the trend of the results for the first query, the study of the electroreduction of CO_2_ has gained significant momentum over the past decade. Most of this research has been carried out at the laboratory scale, including reactor and catalyst design, atomic and molecular modeling, and the kinetics of the electrochemical reactions. In contrast, studies quantifying the economic and environmental impact of ECO2R are relatively scarce yet have slowly gained attention over the last five years. This moderate increase is more pronounced in the case of techno-economic evaluation (series in orange) than for the environmental assessment (series in green).

Based on the results of the search, the rest of this paper proceeds as follows: First, in section experimental advances, we briefly describe current experimental advances and their relation to the scale-up of the technology. Section modeling analyzes the different approaches for the modeling of ECO2R at the industrial scale, which is essential to obtain the data required for the performance, economic, and environmental assessment (section performance assessment). For these two sections, we have analyzed the papers resulting from the economic and the environmental queries and filtered the ones that are out of scope (i.e. contributions that included the keywords “economic” or “environmental” but were not dealing with the techno-economic or environmental assessment of ECO2R processes). The contributions that provide relevant results in terms of production costs and environmental impacts have been used in our assessment and are detailed in the list of references. Finally, we present a summary of opportunities and challenges in ECO2R modeling and evaluation in section perspective and insight.

## Experimental advances

The experimental investigation of ECO2R seeks to quantify the successful conversion of CO_2_ to carbon products using five key figures of merit (FOMs): current density, faradaic efficiency (FE), energy efficiency (applied potential), durability of the equipment, and size of the electrolyzer. These FOMs characterize the performance of the experimental conversion process. However, in order to reach industrially relevant performance, lab-scale phenomena need to be extrapolated to production (industrial)-scale electrolyzers, where the key outcome is product yield. The optimal dimensions of industrial-sized ECO2R electrolyzers are currently unknown due to the lack of connection between the lab-relevant length scale (electrochemical active area) and the total manufactured size of an electrolyzer. Though previous electrolyzer research has revealed chemical reaction trade-offs between longer flow fields and larger stack heights, a formula to calculate the dimensions of ECO2R reactors is yet to be developed. The electrolysis of water is a related electrochemical process that is more commercially mature, but it is unknown if architectures that have shown success with those systems will also be the top performers for ECO2R due to substantial differences in chemistries. Hence, the scaling up of ECO2R will best be done interactively over time to understand the changes in performance that occur over longer scales and guide development in materials and reactor engineering to further improve the large-scale design of the process.

In general, the ECO2R field is challenged by the inconsistent and incomplete reporting of FOMs in publications. This can, in part, be attributed to the dichotomy of advancements researchers are pursuing and differences in laboratory equipment and expertise. Material design focuses on improving the selectivity and activity of the reactions and often reports improvements in partial current densities and faradaic efficiency, whereas reactor engineering and scale-up tackles the challenges in durability, size, single pass conversion, and energy efficiencies of the cell. The future industrial implementation of ECO2R will benefit from complete assessments, where experimental data can be complemented with modeling results, to best represent the trade-offs associated with the scale-up of the technology.

Data for the assessment of ECO2R can come from two primary sources: computational modeling and experimental results. The computational multiphase modeling of ECO2R reactions seeks to understand the underlying physical phenomena using fundamental relationships to explain experimental results and predict performance. Computational models highlight the theoretical limits of different material or chemical combinations and can offer fundamental explanations for phenomena observed in experiments. While good at explaining relationships, models are only as good as their assumptions, relational equations, and the computing power available. The more relationships that are established experimentally, the less computationally intensive models of large systems will become, allowing more large-scale predictions. Previous modeling attempts have mostly been one dimensional, first characterizing materials individually and then characterizing the architectures as a whole (Weng et al., 2019). Recently, models have moved to a two-dimensional space to better account for variations in the feed gas flow (Kas et al., 2021; Yang et al., 2021).

Experimental studies have reported three main ECO2R product types based on the electrocatalyst used: carbon monoxide, formic acid, and multi-carbon products. Due to the differences in the complexity of mechanisms and the phase (liquid or gas) of the products, the three types of ECO2R products require different electrolyzer configurations and have achieved different levels of success. In each product subcategory, however, advancements can be generally categorized as technological advancements which are pushing toward more industrially relevant designs (i.e., favoring high throughputs and low energy demands) and material advancements which are focused on the selectivity and FE of the reaction toward specific products. Herein, a brief description of each electrolyzer design type is given along with the state-of-the-art FOM achieved for each product.

The electrochemical reduction of CO_2_ to carbon monoxide (CO) is mechanistically the simplest reduction reaction, only requiring 2 protons and electrons, and has shown high selectivity and relatively high energy efficiency, leading to a more mature technological state. Research is now focused on achieving the highest current density of CO at the lowest voltage for the longest duration. Liu et al. (2018) recently reported 98% selectivity at approximately 3 V and 200 mA/cm^2^. They held this production for 4,000 hr using an alkaline membrane electrode assembly (MEA) in a zero-gap configuration with an anion exchange membrane (AEM). The authors used a 5 cm^2^ electrode for their work. Future work on CO production will need to replicate similar values on increasingly larger electrodes, and even stacks, while overcoming cell stability issues caused by the consumption of water and subsequent drying out of the membrane.

The production of formate or formic acid is, in some regards, very different from the other two product groups. The conversion of gas to liquid creates unique design constraints for pressure management and mass transfer to and away from the electrocatalyst. Conversely, it also tends to ease subsequent separation stages. The current state of the art in ECO2R to formate uses a MEA with a flowing liquid catholyte to aid in transport (Chen et al., 2020b). Both AEMs and bipolar membranes (BPMs) are being investigated, but BPMs have currently shown reduced crossover and higher durability, bringing them closer to the needs of industrial standards. The reaction to produce formate is challenged by flooding and product crossover which can be addressed with thicker membranes and improved cell design and operational modes. In combination with the liquid catholyte layer, this leads to high overpotentials and low energy efficiencies. Similar to the production of CO, ECO2R to formate has achieved high selectivity at lab scale, and recent work focuses on addressing challenges of industrial scale-up of the process. In a recent step toward large-scale reactors, Chen et al., 2020b demonstrated up to 90% FE to formate at a partial current density of 500 mA/cm^2^ on a significantly larger than typical (25 cm^2^) gas diffusion electrode (GDE) utilizing a BPM in an MEA flow cell. Grigioni et al. (2021) reached a higher current density (930 mA/cm^2^) with an FE of 93% utilizing InP colloidal quantum dot catalysts. Although more selective, their AEM flow cell was only 1 cm^2^ and suffered from flooding during durability testing. Hence, the efficient industrial implementation of ECO2R to formate production will require trade-offs in reactor design between energy efficiency and selectivity to be balanced with overall stability and size.

While CO_2_ reduction to single-carbon products relies on simple, easier to control mechanisms, ECO2R to multi-carbon products has proven more difficult to achieve high selectivities and activities. Copper is the only catalyst to date that yields multi-carbon products in substantial quantities (Hori et al., 1986). The catalyst configuration/facets and different dopants added are used to tailor the products. Adding polymers to the active surface has been a particular focus in the field, as they have been shown the ability to improve selectivity and suppress the competing hydrogen evolution reaction. In their recent report, Chen et al. (2020a) demonstrated this enhanced product selectivity by incorporating a polyamine into the Cu catalyst. They achieved up to 87% FE toward ethylene at −0.47 V vs. reversible hydrogen electrode in a 10 M KOH flow cell. The incorporation of polymers in reactor design for ECO2R to multi-carbon products has also been studied. García de Arquer et al. (2020) reported a partial current density of 1.3 A/cm^2^ toward ethylene utilizing an ionomer incorporated MEA style flow cell. Despite a focus on the reactor scale-up, García de Arquer et al. (2020) still utilized 7M KOH to achieve the lower overpotentials needed. However, lower concentrations of base will be needed to lower overall costs as well as improve cell durability to achieve industrial-scale lifetimes.

Recent work has shown improvements in selectivity and stability when breaking down the reaction into two steps: first performing CO_2_ reduction to CO and then subsequently reducing CO into C_2+_ products, such as ethanol or ethylene (Jouny et al., 2019). This two-step process also eliminates the side reaction of CO_2_ to carbonate species which leads to a loss in CO_2_ and OH- species. Thus, researchers have expanded their studies to explore CO reduction with the aim of producing high value, multi-carbon products, within the value chain of CO_2_ valorization.

To summarize, the field of experimental ECO2R is seeing continuous and increasingly rapid advances. The modeling and assessment of ECO2R have to keep up with these developments, both in terms of process design and operating conditions. This way, they can provide useful information to expand the knowledge of experimentalists further from experimental results, thus providing a valuable feedback loop to accelerate the development and deployment of the technology. For further review of the current experimental advances, we refer the reader to the most up to date review papers and individual studies (e.g. latest reviews by May 2021, not extensive: Tan et al., 2021; Ye et al., 2021; Zhao and Quan, 2021).

## Modeling

**Figure 2:**
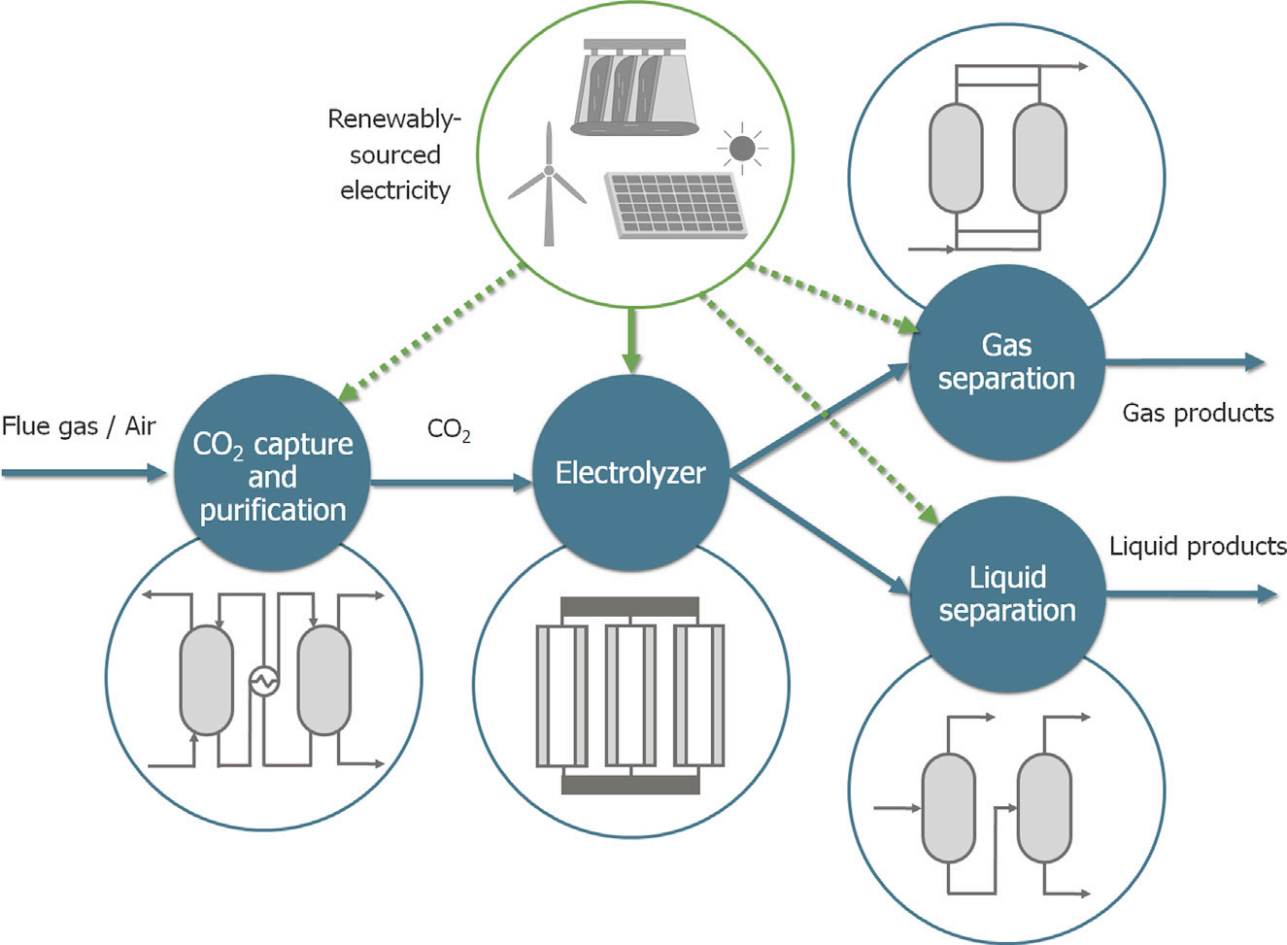
Scheme of the production of chemicals via the electroreduction of CO_2_

Process modeling bridges the data gap from experimental results to the large-scale implementation of the technology and lays the groundwork for the systematic assessment of the implementation of ECO2R. The configuration of an ECO2R process consists of the basic stages depicted in Figure 2. First, CO_2_ is captured and refined either from stationary point sources or from the atmosphere (direct air capture). Next, the one- or two-step electrolysis transforms CO_2_ into products, which have to be separated from the outlet streams. The modeling of carbon capture has been widely studied (Ben-Mansour et al., 2016; Li et al, 2018, 2019; Miller et al., 2014), so in this section, we focus on the modeling of the CO_2_ electroreduction and the subsequent separation units.

Several studies have dealt with modeling electrolysis cells to assess the effect of operating variables on their performance using transport models, heat transfer, and kinetics representations. A number of contributions present models to predict the performance of a solid oxide electrolysis cell for CO_2_ electrolysis. Ni (2010) calculates electrochemical losses including ohmic, activation, and concentration overpotentials, which was then extended with a computational fluid dynamics model to evaluate detailed heat and mass transfer in both the gas channels and the porous electrodes. Xie and Xue (2012) model multi-transport processes of charge, mass, momentum, and energy with detailed surface chemistry for the production of CO. Their results show that high operating temperatures may improve adsorption/desorption rate and mitigate carbon deposition on the catalyst surface. Narasimhaiah and Janardhanan (2013) use the Butler-Volmer equation to evaluate the electrochemical reaction rate at the solid oxide cell. The authors use their model to predict a cost reduction by operating at high potentials and recommend working at conversions below 95% to avoid the formation of coke. Li et al. (2013) present a model for electrode design by coupling an elementary reaction model of CO_2_/H_2_O co-electrolysis with heterogeneous elementary reactions, electrochemical reactions, electrode microstructure, and the transport of mass and charge. Luo et al. (2014) develop a two-dimensional model to analyze the performance and efficiency of said co-electrolysis in a tubular solid oxide electrolysis cell using fluid flow, heat/mass transfer, and electrochemical/chemical reactions and conclude that the reversed water-gas shift reaction promotes the CO_2_ conversion ratio. In the same line, Aicart et al. (2014) build a similar model and perform predictive simulations of partial pressures, current densities, and overpotentials. They conclude that the thermal equilibrium of the cell is strongly dependent on the radiative heat losses. Ren et al. (2018) use a first-principles model with microkinetics details to evaluate the effect of oxygen vacancy locations on the CO_2_ reduction reaction and identify CO desorption as the rate-controlling step. More recent studies have explored other electrolyzer designs. Weng et al. (2018) present a multiphysics model of vapor-fed GDEs for CO_2_ reduction using basic species transport mechanisms, concentration-dependent charge-transfer kinetics, and acid/base kinetics to explore the trade-offs between transport and kinetic trade-offs. They apply the same concepts to build the model for membrane-electrode assemblies (Weng et al., 2019), concluding that the designs with an aqueous feed in the anode present higher current densities than the ones with gaseous feeds at both the anode and the cathode.

These models are built upon lab-scale data and complex mechanics and are hence difficult to translate into the higher-level data needed to make techno-economic and environmental predictions at the process level. The lack of pilot-scale case studies and the accompanying data is another limiting factor for the modeling of an industrial-scale electrolyzer. The extended assumption of a linear scale-up of performance with respect to the size of the electrolyzer may result in unrealistic estimations of the active area of an individual electrolyzer. An oversized electrolyzer model results in a larger electricity consumption and an unrealistic high flux of product, which impacts the subsequent economic and environmental performance estimations. Furthermore, due to the lack of consistency between experimental designs, process modelers have to rely on discrete sampling to overcome the large number of operational variables and design-specific differences between experiments. Without knowledge of the effects of changing system parameters from one experiment to the next, each experiment can only be scaled up in isolation. These limitations result in two main types of electrolyzer models based on their complexity. The first and the most common group is the ones that rely on material and energy balances and stoichiometric relations (Chen and Lin, 2018; De Luna et al., 2019; Jouny et al., 2018; Thonemann and Schulte, 2019) or black box models (Rumayor et al., 2019b). The second and more complex type includes mass transfer effects and the influence of design and operational variables on the selectivity (Orella et al., 2019). Surrogate models have been used to bridge the gap between complex mechanistic models and large-scale assessments in the modeling of carbon dioxide capture (Chung and Lee, 2020; Zhang and Sahinidis, 2013). Similar approaches can be used to model the effect of the main variables of ECO2R on its large-scale design and operational performance. The modeling of the associated separation processes of the gas and liquid outlet streams of the electrolyzer can also be analyzed in terms of model complexity. Most of the existing studies in the literature use simplistic assumptions. For instance, some authors use material and energy balances with fixed separation factors and compositions that are later used for cost or impact parametrization (De Luna et al., 2019; Dominguez-Ramos et al., 2015; Jouny et al., 2018) or empirical models like Sherwood mass transfer correlations to describe separation costs (Orella et al., 2019). These simplified models can be easily applied to different products and operating conditions. However, the separation costs and energy consumption are widely affected by the composition of the output streams from the CO_2_ electrolysis process, which at the same time depends on the corresponding design and operational variables (e.g., current density, overpotential, etc.), yet is often disregarded. Thus, a second group includes more rigorous and comprehensive models for ECO2R with a more detailed modeling of separation stages. These are typically implemented through the use of commercial simulators (Jouny et al., 2018; Thonemann and Schulte, 2019) but have to be product and condition specific.

There exists a clear trade-off between the complexity and accuracy of the model. The assumptions made during the modeling phase have to be carefully selected, as they will substantially affect the results of the assessment stage and consequently influence the decisions made on the implementation of the technology.

## Performance assessment

The performance of ECO2R processes can be assessed in three main areas: technological, economic, and environmental. In addition, a realistic assessment of emerging technologies must take into account their current technology readiness level (TRL) and its expected evolution. However, most works do not include this indicator in their assessment methodology. Chauvy et al. (2019) propose a semi-quantitative method for the selection of CCU products including these three areas. With it, they identify ECO2R to ethanol as one of the promising CO_2_ conversion options for short- to mid-term deployment. An analysis with such a wide focus can be done at the expense of precision. Aiming only to assess the state of technology, Roh et al. (2020) recently published a systematic evaluation procedure for identifying the TRL of CO_2_ utilization technologies and assigned ECO2R a TRL value of 2. Additional works deal with the independent assessment of economic or environmental indicators, which are analyzed in more detail below.

### Economic

Techno-economic analysis (TEA) has been widely used since the first applications of process systems engineering (Pistikopoulos et al., 2021). It is a powerful tool to assess the technical and economic performance of processes that consists of quantifying the design of the process plant and determining the associated costs and revenues of its operation. Many works have implemented TEA on CCU processes (Collodi et al., 2017; Michailos et al., 2019; Pérez-Fortes et al., 2014; Proaño et al., 2020). Recently, Zimmermann et al. (2020) have published detailed guidelines for the TEA of CCU processes. They suggest a four-step method based on LCA standards (International Organization for Standardization, 2006) to unify assessment procedures. Herein, we focus on the specific application of TEA to ECO2R.

**Figure 3:**
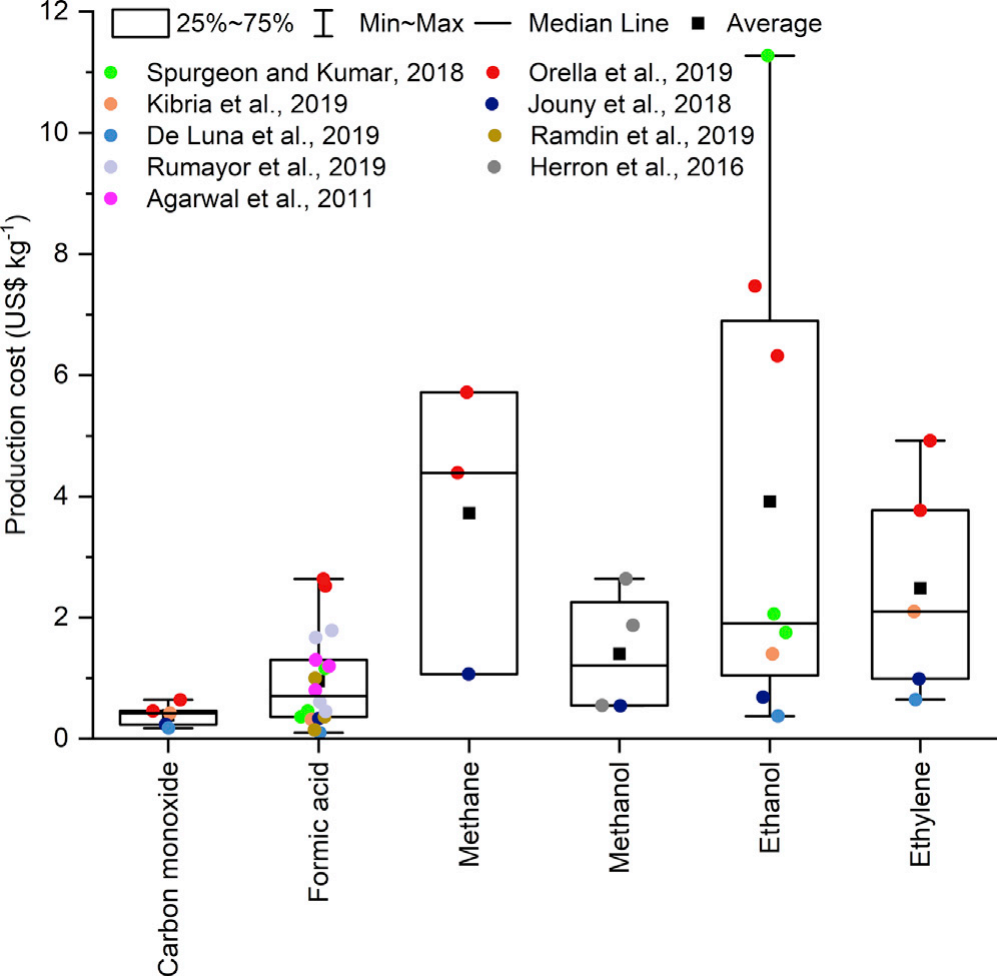
Overview of the results for the direct electrolysis production cost of chemicals from techno-economic analyses in the literature Data source: Agarwal et al. (2011); Herron and Maravelias, 2016; Spurgeon and Kumar, 2018; De Luna et al. (2019); Kibria et al. (2019); Orella et al. (2019); Ramdin et al. (2019); (Rumayor et al., 2019b).

Several studies have carried out techno-economic assessments of the direct electroreduction of CO_2_ to single- and multi-carbon products. Figure 3 summarizes the production costs reported by a set of studies, including results for base case and optimistic scenarios with different assumptions on electricity and CO_2_ feedstock prices. The current market prices for the chemicals under study (assumed to be produced from fossil fuels, data from IHS Markit (2020)) and the results for some statistics calculated from the ECO2R production cost estimations reported in the literature are shown in Table 1. It should be noted that these calculations include the estimates for both current costs and future projections, which could not be isolated due to the moderate volume of data and the different considerations in the optimistic assumptions. Carbon monoxide and formic acid are the two products that are closest to being cost competitive. Indeed, the average electrolyzer-based cost for these chemicals is 2.6 and 1.9 times greater than the US 2019 average market price for CO and formic acid, respectively. The most optimistic future cost projection of the electrochemical production of carbon monoxide is just 17% higher than its current market price and the same value for formic acid is 5 times lower than its market price. However, these calculations and projections vary significantly with the results showing standard deviations of 48% and 81% of the average production cost for CO and formic acid, respectively. Ethylene is next in terms of the gap between the electrolytic production cost and its current market price, with an average ECO2R production cost per kg of $2.49 (425% higher than a market price of $0.58kg^−1^) and standard deviation (74% with respect to the average ECO2R production cost). The statistics for methanol are similar: a reported average ECO2R production cost of $1.4 per kg vs. a market price of 0.26 $/kg leads to an average/market price ratio of 547% and standard deviation/average of 74%. Ethanol, with a market price of $0.48kg^−1^ and an average electrolysis-based production cost of $3.92kg^−1^ shows the highest cost gap with similar values of standard deviation and average production cost. To the best of our knowledge, only two studies reported production costs for methane, which is insufficient to make a thorough assessment, though those reported results are still presented in Figure 3. In general terms, the low gap between the lower bound of the production cost and the market price of the products shows optimistic views toward the future implementation of CO_2_ electroreduction technologies, with carbon monoxide and formic acid being the closest to cost efficiency. However, the high variabilities in the production cost reported by different studies raises the need to further examine the assumptions used for the techno-economic assessment.

**Table 1:** Market price and statistics for the production cost of chemicals via the direct electroreduction of CO_2_ reported in the literature

Products		Carbon monoxide	Formic acid	Methane	Methanol	Ethanol	Ethylene
2019 United States market price^a^ for fossil-based chemicals [$/kg]		0.15	0.50	0.12	0.26	0.48	0.58
Estimated ECO2R production costs from the literature^b^ [$/kg]	Literature average	0.39	0.96	3.72	1.40	3.92	2.48
	Standard deviation	0.19	0.78	2.40	1.03	3.96	1.83
	Minimum	0.18	0.10	1.07	0.54	0.37	0.65
	Maximum	0.64	2.63	5.72	2.64	11.27	4.92

a Source: (IHS Markit, 2020).

b Agarwal et al. (2011); Herron and Maravelias (2016); Spurgeon and Kumar (2018); De Luna et al. (2019); Kibria et al. (2019); Orella et al. (2019); Ramdin et al. (2019); Rumayor et al. (2019b)

**Figure 4:**
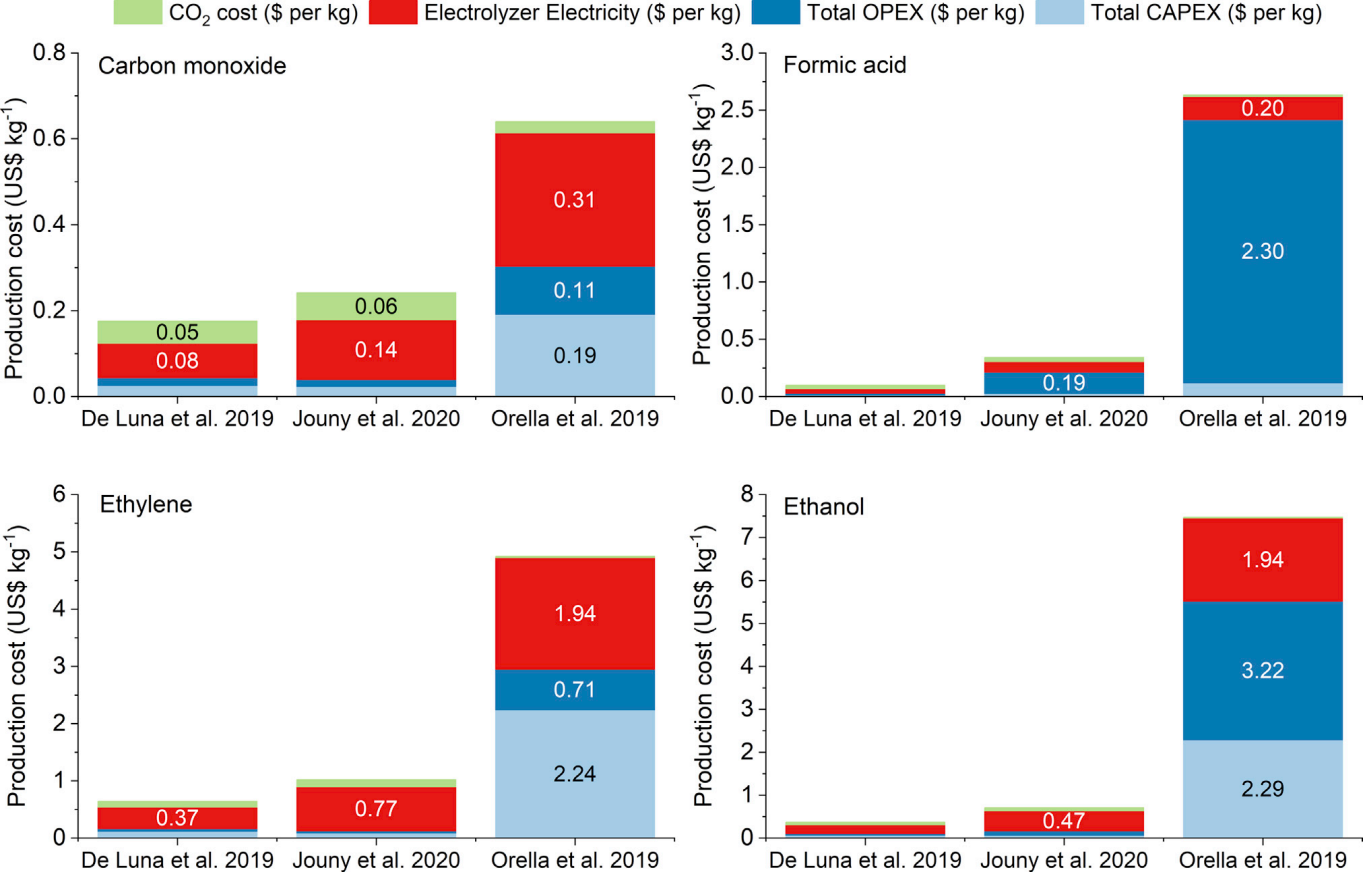
Cost breakdown for the production cost of carbon monoxide, formic acid, ethylene, and ethanol calculated with data from the three TEAs analysed Data source: Jouny et al., 2018; De Luna et al., 2019 and Orella et al., 2019.

Figure 4 depicts the breakdown of the production costs of four chemicals (carbon monoxide, formic acid, ethylene, and ethanol) from three selected references that provide cost breakdown data (De Luna et al., 2019; Jouny et al., 2018; Orella et al., 2019). Assessing the different cost shares reported by each reference for each individual product reveals the effect of different assumptions for cost-related parameters. Regarding the variability of the cost breakdowns, carbon monoxide appears again as the product with the most stable results, due to the maturity of its production via electrolysis. For carbon monoxide, the electricity consumption by the electrolyzer stands out as the main cost driver with an average share of 51% of the total cost and a standard deviation of only 7%. The second largest cost contributor varies depending on the source: 27–30% for CO_2_ feedstock (De Luna et al., 2019; Jouny et al., 2018) vs. 30% for capital costs (Orella et al., 2019). A similar effect is observed for formic acid. While Jouny et al. (2018) and Orella et al. (2019) calculate higher shares for operational costs (57% and 87%, respectively), De Luna et al. (2019) show electricity as the main cost driver (41% of total production cost). While the results for multi-carbon products show more discrepancies concerning cost drivers and their distribution, a general increase in the capital costs can be observed due to the electrolyzer products requiring further separation. Nevertheless, the electricity consumption always represents a significant fraction of the total costs with values ranging from 26% to 78% due to the wide range of electricity price assumptions (0.02 $ kWh^−1^ to 0.10 $ kWh^−1^). Thus, the modeling of electricity rates is critical for the techno-economic evaluation of ECO2R processes.

The parameters that are observed to fluctuate the most and thus are key to providing accurate cost estimations are either technological metrics related to the maturity of the electrolyzer technology (e.g., CO_2_ single-pass conversion, selectivity, power and current densities) or economic (e.g., lifetime, feedstock price, electricity cost). It should also be noted that even though all three works use as a basis the electrolyzer capital costs reported by the hydrogen model (H2A) of the United States Department of Energy (James et al., 2013) for the production of hydrogen via water electrolysis, the resulting capital costs vary significantly due to the different technical values considered for voltage and current density. Lower current density assumptions result in larger electrolyzers, with consequent capital and operational cost increases. These results confirm the importance of unifying technical and economic assumptions and building models that accurately predict the behavior of systems at larger scales. The values for all these relevant technical parameters and costs are currently provided by lab-scale data, simulations, and future projections. Thus, upcoming techno-economic assessments will benefit from adjusting these preliminary TEAs with results for the actual technology developments at pilot plant and industrial scales as they become available.

Aside from these studies on the economic performance of the direct electroreduction of CO_2_ into chemicals, other authors have used techno-economic assessment tools to explore alternative routes for CO_2_ electroreduction. Jouny et al. (2019) applied their techno-economic assessment method (Jouny et al., 2018) to compare the direct route to the two-step conversion process (CO_2_ reduction into CO, which is then reduced into acetic acid or ethylene), concluding that even if capital costs are increased, electricity costs are significantly reduced together with a performance increase, due to increased product selectivity and hence lower separation costs. Similarly, Li et al. (2016) explore splitting the CO_2_ reduction process into CO reduction and the Fischer-Tropsch process and conclude that the economic competitiveness of the resulting product with respect to petroleum-based products relies on simultaneous improvement of both the technologies used, decreasing the likelihood of its realization, as Fischer-Tropsch process is a very mature technology. Another combined alternative is that proposed by Na et al. (2019). They tested the coupling of carbon dioxide reduction reactions with organic oxidation to improve the economic feasibility of the technology and report better economic performance with respect to the traditional ECO2R processes, using market price for formic acid, n-propanol, acetaldehyde, allyl alcohol, glycolaldehyde, and ethylene glycol as a reference. In the same vein, Verma et al. (2019) find the co-electrolysis of CO_2_ and glycerol to be a promising alternative for lowering electricity consumption up to 53%.

Additionally, some works explore the integration of the technology with process and energy systems. This is the case in Herron and Maravelias (2016), who analyzed the process economics for a solar refinery that converts CO_2_ into methanol using a photovoltaic-powered electrolyzer. Their method provides targets for the performance of electrocatalysts and solar electricity generation to render the process economically competitive and conclude that the solar-powered electrocatalytic reduction is ultimately limited by the price of solar electricity. Conversely, the work by Ioannou et al. (2020) explores the use of mathematical optimization to find hybrid (fossil and CO_2_ based) routes for the production of ethylene. While they conclude that the thermochemical route is currently economically and environmentally more efficient, they also determine that higher electrolyzer efficiencies would increase the viability of the electrosynthesis route. The hybrid route is economically more expensive (by 30%) but environmentally more efficient (showing 54% and 29% decrease in the environmental impacts on ecosystems quality and resources, respectively).

All these works provide useful techno-economic assessment tools to estimate the costs of CO_2_ reduction and identify technical and economic targets for its cost competitiveness. However, there is a need for unified cost scenarios, e.g., current, near future (2030), and long term (2050), as well as more detailed modeling of electricity prices in low-carbon electricity grids. For instance, more robust process models would allow for more realistic process designs, which could reduce the uncertainties associated with capital and operating cost estimations. Indeed, most of the CO_2_ electrolysis experimental works have been carried out at laboratory scale, e.g., relative low current density and low energy efficiency. Thus, there is a need for a better understanding of the operation of CO_2_ electrolysis at industrial scales, e.g., higher current densities and using CO_2_ streams with impurities (SOx, NOx, etc.). On the other hand, the iterations between CO_2_ electrolysis processes and electricity markets require a better understanding. For example, the variability of electricity prices increases as the share of wind and solar PV power in the energy mix increases, which could require a more flexible operation of the electrolyzers to take advantage of the electricity price dynamics and face cost variabilities over time. Thus, the value of CO_2_ electrolyzer flexibility in view of dynamic electricity prices requires a better understanding.

### Environmental impact

The recent publication of reviews and guidelines about the application of LCA to CCU has revealed the emergence of a body of work on the adaptation of current LCA practices to the new challenges that CO_2_-based processes pose. In this section, we refer to some of these general studies and inspect their conclusions related to the environmental impacts of ECO2R. Artz et al. (2018) made an extensive review of catalysts and their impact on the LCA of CO_2_ conversion to identify opportunities to use CO_2_ as a feedstock and thus avoid the utilization of fossil resources. The authors compare the electrochemical conversion of CO_2_ and methanol to dimethyl carbonate and alternative processes for its production. They state that breakthrough improvements in the process design would be required for the electrochemical route to be environmentally beneficial. Koj et al. (2019) performed a review of 32 LCA studies on Power-to-X revealing a lack of transparency on technological and methodological assumptions, especially dealing with multi-functionality, for processes that yield several products. The authors also highlight the source of electricity as a crucial driver of the environmental impact. Very recently, a similar study focused on 52 peer-reviewed articles that dealt with LCA and CO_2_-based chemical production (Thonemann, 2020). When comparing CO_2_-based paths for the production of formic acid to the conventional process, hydrogenation performs better in most indicators, but the electrochemical route shows promising results in terms of impacts on climate change and human health. All of these reviews stress the different methodological and technical choices found in the literature and the need to unify criteria in pursuit of comparability. Hence, Müller et al. (2020) define a systematic selection of the functional unit and system boundaries based on the final use of the CCU product (as energy storage; or chemicals, materials, fuels, and others) and the similarities in chemical structure and composition to the traditional product to which it is compared. They also offer modeling assumptions to deal with multi-functionality in CCU, as well as options to bridge data gaps.

Previous CCU research has addressed the hydrogenation of CO_2_ into formic acid with hydrogen supplied by water electrolysis (Hoppe et al., 2018; Pérez-Fortes et al., 2016; Sternberg et al., 2017). However, there are only a limited number of studies on the LCA of the direct electrochemical reduction of CO_2_ into chemicals that provide detailed impact breakdowns in terms of feedstocks, process stages, and energy sources. Dominguez-Ramos et al. (2015) published one of the first LCA studies on ECO2R, with formate as the main product. Although the authors report results for greenhouse gas emissions 10 to 170 times higher than the conventional process under the current state of technology at the time, they find some encouraging results for a very optimistic future scenario (100% FE, extractive distillation, and a solar photovoltaic-powered electrolyzer), with greenhouse gas emissions 41% lower than the conventional process. In former works, they evaluate the environmental competitiveness of the production of formic acid by ECO2R (Rumayor et al., 2018) and the effect of cathode lifetime (Rumayor et al., 2019a). They later included the influence of time to assess the evolution of the impact and the influence of energy systems on the environmental performance of the process (Aldaco et al., 2019).

Thonemann and Schulte (2019) analyzed the critical matter of scaling up emerging technologies and proposed a methodology to apply LCA to evaluate the environmental impact of future ECO2R processes. The authors tested their method on the ECO2R to formic acid through the definition of different scale-up scenarios: (1) laboratory data, (2) the best-case estimate assuming ideal conditions, and (3) scale-up with more realistic technical assumptions, where they test different reactor design scale-ups. The resulting global warming impacts (GWIs) of batch reactor and three-compartment cell (TCC) configurations are higher than those of the flow-through reactor (FTR) scale-up. In the recent work of Kibria Nabil et al. (2021), the authors presented a comparative LCA of one- and two-step electrochemical conversion of CO_2_ into eight bulk chemicals (carbon monoxide, formic acid, methane, methanol, ethylene, ethanol, n-propanol, and acetic acid). They reported lower carbon intensity in the two-step route, due to the avoidance of carbonate formation, and found that syngas, ethylene, and n-propanol were the most compelling products in terms of GWI.

**Figure 5:**
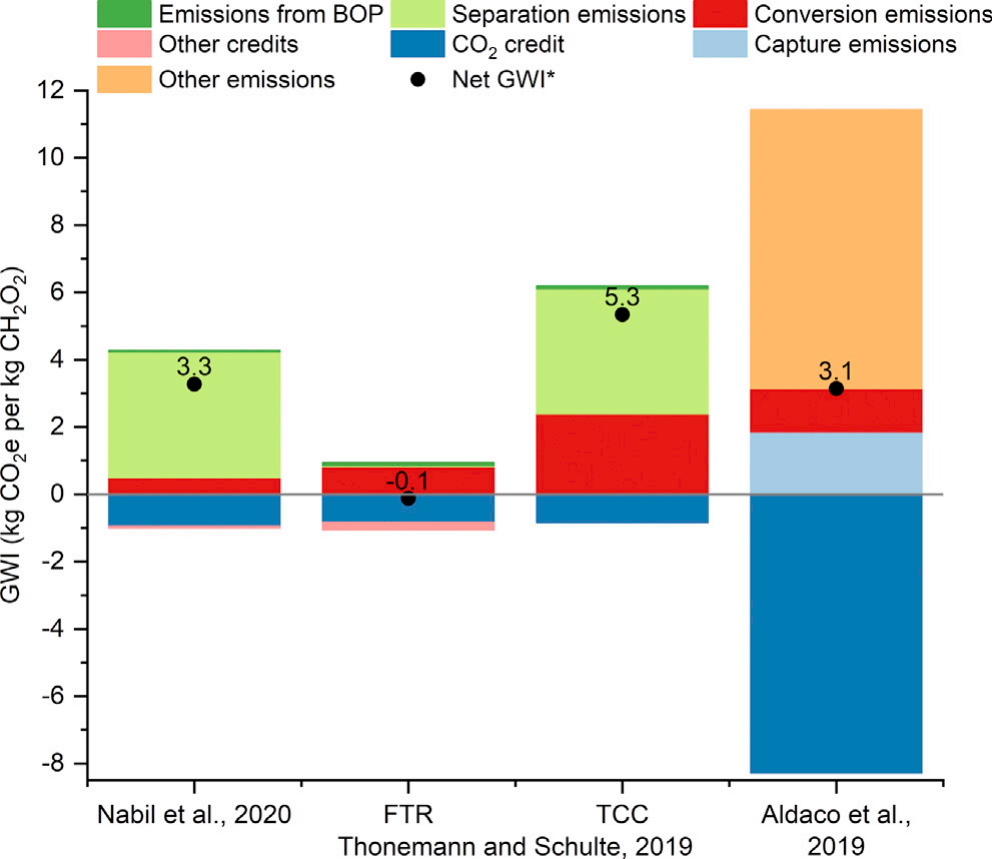
Global warming impact (GWI) breakdown in kg CO2e per kg of formic acid reported by the three references analysed Data source: Aldaco et al., 2019; Nabil et al., 2020; Thonemann and Schulte, 2019. Credits for avoided CO_2_ emissions and by-products (other).

Figure 5 shows the breakdown of the GWIs (kg CO_2_ eq per kg of product) for direct ECO2R to formic acid reported in these last studies (Aldaco et al., 2019; Nabil et al., 2020; Thonemann and Schulte, 2019). It should be noted that Aldaco et al. (2019) report results for the aggregated process emissions instead of a conversion, separation and balance of plant breakdown, represented in “other emissions”. Also, it is important to note that different assumptions are made regarding the distribution of the impacts based on the categories “CO_2_ credit” and “conversion emissions”. Thonemann and Schulte (2019) provide only a positive “CO_2_ credit” where capture emissions were previously subtracted. For this reference, we have selected the scale-ups that they claim to be more realistic with current technology advancements (TCC) and the design with assumptions that are more realistic for future applications (FTR). The average GWI for the current estimates (excluding FTR) is 2.94 kg CO_2_ per kg of formic acid, while the optimistic solution of Thonemann and Schulte (2019) is the only one where the credits exceed the impacts, due to a dramatic reduction in the emissions from the separation stages, which are the main source of GWI in current estimates. Hence, the energy intensity of purification processes becomes a crucial variable to control the emissions of ECO2R.

Table 2 lists the main LCA modeling assumptions made by each reference. There seems to be an agreement on using consequential cradle-to-gate analysis and ecoinvent as the database for life cycle inventory. However, the life cycle impact assessment (LCIA) methodology selected differs or is not specified. Some studies in other fields prove that the resulting impacts are sensitive to the impact assessment method (Bovea and Gallardo, 2006; Renou et al., 2008; Zhou et al., 2011), highlighting the need for unified criteria. In this sense, Müller et al. (2020) recommend the use of CML (Institute of Environmental Sciences, University of Leiden) in its most recent version for CCU applications. Nevertheless, further research should be performed to determine which method is more suitable for the assessment of ECO2R in particular. The number of indicators analyzed is scarce. While GWI is a widely used metric by the LCA community, future studies should tackle the inclusion of a combination of midpoint and endpoint indicators to extend the reach of the analysis. These assessment divergences stack with the different assumptions made in the modeling stage when defining the inventory, preventing the comparability of different studies. Another major concern to explore in future research is the assessment of different products and routes and their integration with current fossil technologies.

**Table 2:** Assumptions for the three LCAs studied

Reference	Nabil et al. (2020)	Thonemann and Schulte (2019)	Aldaco et al., (2019)
Approach	n.s.	Consequential	Dynamic
Functional unit	1 kg of FA	1 kg of FA	1 kg of FA
Scope	Cradle-to-gate	Cradle-to-gate	Cradle-to-gate
Software	GaBi Professional software	openLCA 1.7.4	GaBi Professional software
Database	ecoinvent 3.5	ecoinvent 3.4	ecoinvent 3.3
LCIA method	n.s.	ILCD 1.0.8	CML 2016

∗n.s.: not specified.

Data source: Aldaco et al., 2019; Nabil et al., 2020; Thonemann and Schulte, 2019.

## Perspective and insight

The electroreduction of CO_2_ is emerging as an attractive alternative technology compared to fossil-based chemicals, opening opportunities in many different sectors. However, the maturity of the technology and the required shift from fossil-based technologies pose some challenges that will have to be addressed for the extensive adoption of CO_2_ reduction to chemicals and fuels. In this context, process, techno-economic, and environmental models are analytical tools that can provide insights into the research and development needs for the industrial deployment of electroreduction of CO_2_. This section will examine some of these challenges and opportunities to identify the areas for improvement and define pathways toward the industrial implementation of the technology.

Since many technologies fail in the transition from benchtop to industrial scale, developing a deeper understanding of the physical and energetic scaling relationships of ECO2R systems will be essential to designing optimized ECO2R processes at scale. An efficient bidirectional feedback loop between early industrial adopters and experimental research will be necessary, as it will provide critical data for systems engineering and reactor design to further optimize this technology. A successful example of this can be seen in the work by Guo and Sun (2020), where the authors use the analysis from Jouny et al. (2018) to calculate the competitiveness of a newly developed catalyst. Here, data availability, quality, and the inclusion of uncertainty should be targeted.

The adoption of ECO2R will also require a multi-scale integration effort by connecting the advances that are currently being made at different scales: laboratory (Huang and Hu, 2018; Zhao et al., 2020), plant (van Bavel et al., 2020), and supply chain (Leonzio et al, 2019, 2020). ECO2R processes can be enhanced via integration with other CO_2_ conversion methods such as photocatalytic, CO_2_ polymerization, biohybrid, and molecular machine technologies. Hybrid solutions that combine electrolysis and traditional synthesis to take advantage of existing facilities and equipment provide one promising avenue toward gaining experience with ECO2R technologies that limit capital costs and hence have the possibility to provide a smooth transition away from current fossil-based technologies. In this light, holistic approaches will be needed to model and assess both components and entire ECO2R processes, and key performance indicators should be unified to ensure comparability among processes or products.

The modeling and assessment of ECO2R could also benefit from game theory approaches to analyze the interaction of the multiple stakeholders involved in the process of adoption of the technology (i.e. private companies, academic and research institutions, local government). In particular, policy-makers become a pivotal actor in the adoption of ECO2R through carbon taxes and incentives to clean technologies. Proposed strategies to manage the decarbonization transition (Bataille et al., 2018) have to be complemented with specific policies on the provision of CO_2_ emission-free baseload electricity (Schmidt, 2021). Transition plans should be tailored to the individual industries and respond to changing policy support needs as technology develops (Binz et al., 2017). Furthermore, policies should be designed to deal effectively with the associated investment risk (Egli, 2020). Sector coupling with renewable energy (using low-cost or curtailed renewable electricity in Power-to-X applications) will be crucial for the success of ECO2R. First, the adoption of renewable power in the chemical industry, e.g., for the electrochemical reduction of CO_2_ to chemicals, could facilitate the integration of ultra-high wind and solar photovoltaic energy shares into broader energy systems (Chu et al., 2017; Whipple and Kenis, 2010). The use of otherwise curtailed renewable power could improve the economics of renewable power plants in very high renewable power systems and open new markets for renewable power. On the other hand, the integration of renewable power into the chemical sector could help to decarbonize the chemical industry, which is considered a difficult-to-decarbonize energy sector (Davis et al., 2018; Hepburn et al., 2019). However, there is a need for a better understanding of the operational and economic aspects of integrated energy systems with Power-to-X applications. For example, most of the existing studies in the literature are based on a flat price for renewable electricity (De Luna et al., 2019; Jouny et al., 2018; Orella et al., 2019). However, the integration of Power-to-X pathways with wholesale or retail electricity markets would likely involve volatility in electricity prices. Thus, the flexibility of ECO2R processes requires additional study to understand the design trade-offs between reduced capital and electricity costs. Additionally, the identification of the most cost-effective pathways as well as cost and technology targets could facilitate the early adoption of these technologies. In summary, there is a need for more comprehensive analyses of ECO2R pathways in view of high renewable energy systems, technology readiness levels, and future electricity markets. Indeed, the appropriate use of modeling, TEA, and LCA tools has the potential to guide experimental ECO2R research, reducing production costs, and thereby accelerate the industrial adoption of ECO2R.

### Limitations of the study

No computational analysis is presented. Limitations are related to the search engine. To further understand the limitations of the literature sampling and reviewing procedure, please, refer to the Methodology section.
